# 

**Published:** 1900-06

**Authors:** 


					CONTENTS. i I
Presentation to Mr. L. M. Griffiths
... 97
A Retrospect of a Second Series
of Fifty Consecutive Intra-
Abdominal Operations.
By James
Swain, M.S., M.D. Lond., F.K.C.S.
Eng
103
Cases of Acute Intestinal Obstruction
Treated by Operation.
By W. H.
Harsant, F.R.C.S.
118
Abdominal Hysterectomy for
Fibroids of the Uterus, with
Retro-Peritoneal Treatment of
the Stump, and Notes of two
By W. C. Swayne, M.D.
Lond.
.. 123
Two Cases of the Sporadic Form
of Epidemic Cerebro - Spinal
Meningitis.
By J. Michell
Clarke, M.A., M.D. Cantab.,
t.R.C.P. Lond
128
Swedish Medical Gymnastics in
Chronic Diseases of the Heart.
By E. Chkistokferson
134 i
Hote on a Pre-Exanthematous Sign
of Measles.
By P. WATSON
Williams, M.D. Lond.
139
Progress of the Medical Sciences:
Medicine.
R. Shingleton Smith, ;
M.D.
? !
Surgery.
C. A. Morton, F.R.C.S.
146
ObJ?tetcic3.
Walter C. Swaynk,
M.D.
191
Reviews of Books
155
Rotes on Preparations for theJSick
Meetings of Societies
The Library of tho Bristol Medico-
Chirurgical Society 182
Obituary
183
Local Medical Notes  186
Scraps
191

				

